# Psychosocial stress moderates the relationship between cerebrospinal fluid lactate dehydrogenase and the duration of untreated psychosis in first-episode psychosis

**DOI:** 10.3389/fpsyt.2024.1327928

**Published:** 2024-02-15

**Authors:** Eloi Giné-Servén, Ester Boix-Quintana, Eva Daví-Loscos, Sandra Cepedello, Lara Moreno-Sancho, Marta Niubó, Rebeca Hernández-Antón, Manuel J. Cuesta, Javier Labad

**Affiliations:** ^1^ Department of Psychiatry, Hospital Universitario de Navarra, Pamplona, Spain; ^2^ Instituto de Investigación Sanitaria de Navarra (IdiSNA), Pamplona, Spain; ^3^ Department of Mental Health and Addictions, Consorci Sanitari del Maresme, Mataró, Spain; ^4^ Translational Neuroscience Research Unit I3PT-INc-UAB, Institut de Innovació i Investigació Parc Taulí (I3PT), Institut de Neurociències, Universitat Autònoma de Barcelona, Sabadell, Spain; ^5^ Centro de Investigación Biomédica en Red de Salud Mental (CIBERSAM), Madrid, Spain

**Keywords:** psychosis, duration of untreated psychosis, stress, cerebrospinal fluid, lactate dehydrogenase, glucose

## Abstract

**Introduction:**

Previous research has shown that lower lactate dehydrogenase (LDH) concentrations in cerebrospinal fluid (CSF) are associated with longer prodromal symptoms in first-episode psychosis (FEP). We aimed to study whether there is a relationship between the duration of untreated psychosis (DUP) and LDH and other CSF biomarkers in FEP and whether stressful life events moderate this association.

**Methods:**

Ninety-five inpatients with FEP and with less than 6 weeks of antipsychotic treatment were included in the study. All participants were informed about the nature of the study, which was approved by the local ethics committee, and signed an informed consent form. A lumbar puncture was performed at index admission (baseline) to measure CSF parameters (glucose, total protein, LDH). The DUP was assessed with the Quick Psychosis Onset and Prodromal Symptoms Inventory (Q-POPSI). Stressful life events (SLEs) in the previous 6 months were assessed with the List of Threatening Experiences. We dichotomized the SLE variable into having experienced at least one SLE or no experience of SLEs. Statistical analyses were performed with SPSS v. 25.0. Total protein and LDH concentrations were natural log transformed (ln) to reduce skewness. Multiple linear regression analyses were conducted to explore the association between the DUP and CSF parameters (considered the dependent variable). Age, sex, DUP and SLEs were considered independent variables. We tested the DUP by SLE interaction. Significant interactions were included in the final model. The threshold for significance was set at p<0.05.

**Results:**

Fifty-four FEP patients (56.8%) reported an SLE in the previous 6 months. There were no significant differences in the DUP between patients with or without SLEs. There were no significant differences in CSF biomarkers between the SLE groups. In the multiple linear regression analyses, we found a significant DUP by SLE interaction effect on CSF LDH concentrations (standardized beta= -0.320, t= -2.084, p= 0.040). In patients with SLEs, a shorter DUP was associated with higher CSF LDH concentrations and vice versa. No significant associations were found between the DUP or SLEs and other CSF biomarkers (glucose, total proteins).

**Conclusions:**

Our study suggests that psychosocial stress moderates the relationship between the onset of psychosis and CSF biomarkers related to bioenergetic systems.

## 1 Introduction

The onset of psychotic illness typically occurs in late adolescence or early adulthood. Approximately 3% of the population experience a first episode of psychosis (FEP), and its clinical evolution tends toward chronicity (1). In recent decades, a substantial body of evidence has emerged that highlights the importance of early intervention for psychotic disorders, with a specific focus on reducing the duration of untreated psychosis (DUP; the time from the onset of psychotic symptoms until first treatment) (2). These studies have revealed that a longer DUP is linked to more severe positive symptoms (3, 4), negative symptoms (3–5), and impairment of functioning (3, 4). Furthermore, certain studies have indicated higher relapse rates or poorer responses to antipsychotic medications in individuals with a longer DUP (5). The development of psychotic disorders is probably linked to abnormalities in brain development during early life, which then interact with abnormal pathophysiological processes during puberty, ultimately culminating in the onset of psychotic illness (6, 7). Aberrant synaptic pruning (7), sensitization of the dopaminergic system (8), and neurobiological adaptations to chronic N-methyl-D-aspartate receptor dysfunction (9) are possible pathophysiological mechanisms that produce limited neurodegeneration early in the course of psychotic illness (10). The question of whether psychosis itself has a neurotoxic effect is still a topic of debate (11), and there are several hypotheses regarding how untreated psychosis might influence brain function. Dopaminergic hyperactivity, resulting in a gradual decrease in regional brain volumes (12), and oxidative damage due to persistent catecholaminergic activity and prolonged activation of the hypothalamic–pituitary–adrenal axis (13) are potential explanations for the neurotoxic effect of chronic psychosis. Neurotransmitter dysfunction could be a consequence of cell membrane pathology in individuals with schizophrenia (14). Certain areas of the brain, such as the temporal regions, may be particularly susceptible to the impacts of a longer DUP (2).

On the other hand, the interplay between stress and vulnerability has been recognized as a factor in the development of psychotic disorders (15–17). Previous studies suggest that early life stress, particularly childhood trauma (18, 19), is a risk factor for psychotic disorders. Several studies have also reported increased perceived stress in individuals at clinical high risk of psychosis (CHR) or with psychotic disorders, measured using self-report questionnaires (20–22) or electronic sampling methods (23). Regarding hypothalamic-pituitaryadrenal (HPA) axis measures, previous longitudinal studies suggest that diurnal cortisol (24, 25), an increased cortisol awakening response (CAR) (26), and a higher stressor-cortisol concordance (27) are found in CHR individuals who will later develop psychotic symptoms. However, in first episode psychosis (FEP) patients, a blunted CAR has been described (28–30), suggesting that biological markers of stress response might be influenced by the stage of psychotic illness. Consistent with this, previous studies exploring stress responsiveness with the Trier Social Stress Test in CHR and FEP patients (31) found differences in autonomic measures between groups (elevated heart rate and blood pressure were observed in FEP patients), suggesting that the stage of illness contributes to variations in the psychobiological stress response.

The experience of stress – psychosocial, biological, or both – influences neurobiology via a range of pathways, including immune, stress/endocrine, redox, and metabolic systems (32). Neurobiological substrates of the aberrant stress response to HPA axis dysregulation, disruption of inflammatory processes, increased oxidative stress, gut dysbiosis, and altered brain signaling provide mechanistic links between environmental risk factors and the development of psychotic symptoms (33). The importance of stress in triggering the onset and relapse of psychosis is now widely acknowledged. In the last decade, the major research challenge in this area has been to clarify the biological mechanisms underlying the interplay between stress and the onset of psychosis. A clear emphasis of this research is the examination of the biological systems implicated in the stress response. Stress biomarkers seem to have substantial potential as predictors of psychosis and clinical outcomes and may serve as ideal targets for the development of new therapeutic treatments for psychosis (34). It is also important to balance the timing of the stress response including the immune and inflammatory mechanisms within diathesis-stress models of psychosis, as acute stress and inflammation is usually useful and adaptive, turning off once resolved, whereas chronic stress and inflammation is typically maladaptive and associated with tissue-specific or systemic pathology (28).

Our previous studies have reported associations between cerebrospinal fluid (CSF) biomarkers that involve bioenergetics systems (e.g., lactate dehydrogenase [LDH] and glucose) and the clinical expression of first-episode psychosis, including an association between lower CSF LDH concentrations and prodromal symptoms (35). LDH plays a crucial role in brain function by participating in the conversion of lactate to pyruvate, a process essential for energy metabolism in neurons; this enzyme helps maintain the delicate balance between glycolysis and oxidative phosphorylation, ensuring sufficient energy production to support various neurological functions (35). As lower LDH concentrations in CSF have been associated with prodromal symptoms, and these symptoms might be associated with a longer DUP, we hypothesized that the DUP would be associated with lower LDH concentrations in CSF. Additionally, we aimed to explore whether stressful life events moderate this association.

## 2 Methods

### 2.1 Study design and participants

Patients experiencing FEP admitted to acute inpatient units (adult or child and adolescent units) at the Department of Mental Health at the Hospital of Mataró between June 1st, 2018, and March 31st, 2021, were invited to participate in the study. FEP was defined as new-onset disorganized behavior accompanied by delusions or hallucinations, not caused by drugs, that met the DSM-IV criteria for a psychotic disorder (schizophrenia, bipolar disorder or unipolar major depression with psychotic features, schizophreniform disorder, brief psychotic disorder, delusional disorder, psychotic disorder not otherwise specified). Patients were excluded if they had (1) positive symptoms of psychosis lasting more than 6 months, (2) received treatment with antipsychotics for more than 6 weeks, (3) a past history of positive symptoms of psychosis, (4) a previous diagnosis of intellectual disability (IQ < 70), or (5) active medical or neurological diseases that could explain the current symptoms. Ninety-five FEP patients participated in the current study. This sample partially overlapped with the sample in a previous study (36) that explored different hypotheses.

The study was approved by the local ethics committee (Hospital of Mataró, Barcelona, Spain). All participants were informed about the nature of the study and provided written informed consent to participate in the study.

### 2.2 Clinical assessment during hospital admission

During the first week of hospital admission, all patients underwent psychiatric and neurological evaluations. Two trained attending psychiatrists carried out diagnostic interviews using the Structured Clinical Interview for DSM-IV-TR (SCID-I) (36) for individuals ≥18 years old, the Schedule for Affective Disorders and Schizophrenia for school-age children, and the Present and Lifetime version (K-SADS-PL) (37) for individuals <18 years old.

The onset of prodromal and psychotic symptoms was assessed retrospectively by means of a semi structured interview with a specific *ad hoc* inventory (Quick Psychosis Onset and Prodromal Symptoms Inventory [Q-POPSI]). The duration of untreated illness (DUI) and duration of untreated psychosis (DUP) were calculated. A full description of the Q-POPSI has been provided elsewhere (35).

Psychopathology at admission was assessed using three psychometric scales. The Positive and Negative Syndrome Scale (PANSS) (38) was used to assess positive, negative and general psychopathology symptoms. The Young Mania Rating Scale (YMRS) (39, 40) was administered to assess manic symptoms. The Hamilton Depression Rating Scale (HAM-D) (41, 42) was also administered to assess depressive symptoms.

Stressful life events (SLEs) in the previous 6 months were assessed with the List of Threatening Experiences (43); individuals were categorized into groups according to whether they had experienced at least one SLE.

Baseline functioning was assessed at admission in the acute inpatient unit using the Global Assessment of Functioning Scale (GAF) (44) for individuals ≥18 years old and the Children’s Global Assessment Scale (C-GAS) (45) for individuals <18 years old.

A description of the clinical and biological assessments is provided in **Table 1**.

**Table 1:** Clinical characteristics of the sample.

Age, mean (SD), years	35.0 (15.5)
Female sex, N (%)	39 (41.1%)
Smoking, N (%)	50 (52.6%)
Cannabis use (abuse or dependence), N (%)	47 (49.5%)
Alcohol use (abuse or dependence), N (%)	29 (30.5%)
First degree family history of psychiatric disease, N (%)	42 (44.2%)
Previous stressful life events, N (%)	41 (43.2%)
Duration of untreated illness, mean (SD), days	172.0 (260.2)
Duration of untreated psychosis, mean (SD), days	37.9 (51.7)
Treatment during hospital admission, N (%) Atypical antipsychotics Typical antipsychotics Mood stabilizers Electroconvulsive therapy	95 (100%) 8 (8.4%) 47 (49.5%) 1 (1.1%)
PANSS, mean (SD) Total score Subscale scores: Positive factor Negative factor Disorganized/concrete factor Excited factor Depressed factor	82.6 (20.1) 14.4 (3.5) 12.1 (7.0) 8.8 (2.9) 11.6 (4.1) 8.3 (3.6)
YMRS score, mean (SD)	26.8 (11.3)
HAM-D score, mean (SD)	22.7 (9.8)
GAF score, mean (SD) On admission At discharge	30.0 (6.7) 62.6 (9.6)
Cerebrospinal fluid variables	
Parameters, mean (SD)	
Cells (cells/μL) Glucose (mg/dL) Total protein (mg/dL) LDH (U/L 37C)	1.7 (1.7) 65.4 (6.3) 31.4 (11.4) 27.8 (15.2)
Abnormal cerebrospinal fluid test results, N (%)	9 (9.4%)
Pleocytosis (>5 white blood cells/μL in the CSF) Increased protein concentration (>45 mg/dL)	3 (3.1%) 6 (6.3%)

SD, standard deviation; PANSS, Positive and Negative Syndrome Scale; YMRS, Young Mania Rating Scale; HAM-D, Hamilton Depression Rating Scale; GAF, Global Assessment of Functioning; LDH, lactate dehydrogenase; CSF, cerebrospinal fluid.

### 2.3 Routine CSF studies

All patients underwent lumbar puncture by a neurologist, either at the emergency department before admission or at the inpatient unit during the first week of admission. All participants were studied for NMDAR-Abs and GAD65-Abs in their CSF while they participated in another study dealing with autoimmune encephalitis in psychosis (46). None of them had a diagnosis of encephalitis.

CSF samples were examined to determine blood cell counts (ref <5/µL) with on a Sysmex XN 1000 (Sysmex Corporation, Japan) automatic counting versus manual counting chamber. Quantitative determination of glucose, total protein and lactate dehydrogenase levels was performed on a COBAS INTEGRA (Roche Diagnostics, Spain) using the hexokinase method (glucose), the Biuret method (total protein) and the lactate to pyruvate reaction in N-methylglucamine buffer (lactate dehydrogenase), respectively. The sensitivity of the assays was 4.35 mg/dL for glucose, 4 mg/dL for total proteins, and 10 (IU/L) for LDH. The intra-assay and interassay coefficients of variation were 0.8% and 2.5% for glucose, 2.25% and 5% for total proteins, and 1.29% and 1.7% for LDH, respectively.

### 2.4 Statistical analysis

All data analyses were performed using IBM SPSS for Windows, version 25.0 (IBM Corporation, USA). Continuous CSF parameters (glucose, total protein, LDH levels) were natural log transformed (ln) to reduce skewness and achieve normalization before conducting parametric analyses and regression analyses. We used a dichotomous variable for SLEs, according to whether individuals had experienced at least one SLE.

Spearman and Pearson correlation analyses were used to explore associations between variables. Partial correlation analyses were used to adjust for covariates. To compare continuous data between groups (e.g., FEP patients with or without SLE), Student’s t test was used. The statistical significance threshold was set at p value <0.05 (two-tailed).

Regarding the cell count in the CSF, as only 3 patients had pleocytosis (>5 white blood cells/μL), we decided not to explore associations between this CSF measure and symptoms or diagnoses in the multivariate analyses. Therefore, all hypotheses involving CSF variables considered only the three parameters treated as continuous variables (total protein, LDH and glucose levels).

Multiple linear regression analyses were used to test the hypotheses and explore the association between the DUP and CSF biochemical parameters. In these analyses, we developed three independent equations (one for each CSF parameter [glucose, total protein, LDH levels]; CSF parameters were considered the dependent variable). Age, sex, DUP and SLEs were considered independent variables. We tested the DUP by SLE interaction. Significant interactions were included in the final model. The significance threshold was set at p<0.05.

## 3 Results

Demographic, clinical and biochemical data of the sample at the baseline assessment are shown in **Table 1**. Only a small proportion of FEP patients (<10%) had abnormal CSF findings.

Fifty-four patients (56.8%) reported an SLE in the previous 6 months. There were no significant differences in the DUP between patients with and without SLEs (40.2 ± 60.6 vs. 34.9 ± 37.5, p= 0.625). There were no significant differences in CSF biomarkers between the SLE groups.

No significant correlations were found between CSF biomarkers (glucose, total proteins, LDH).

**Figure 1:**
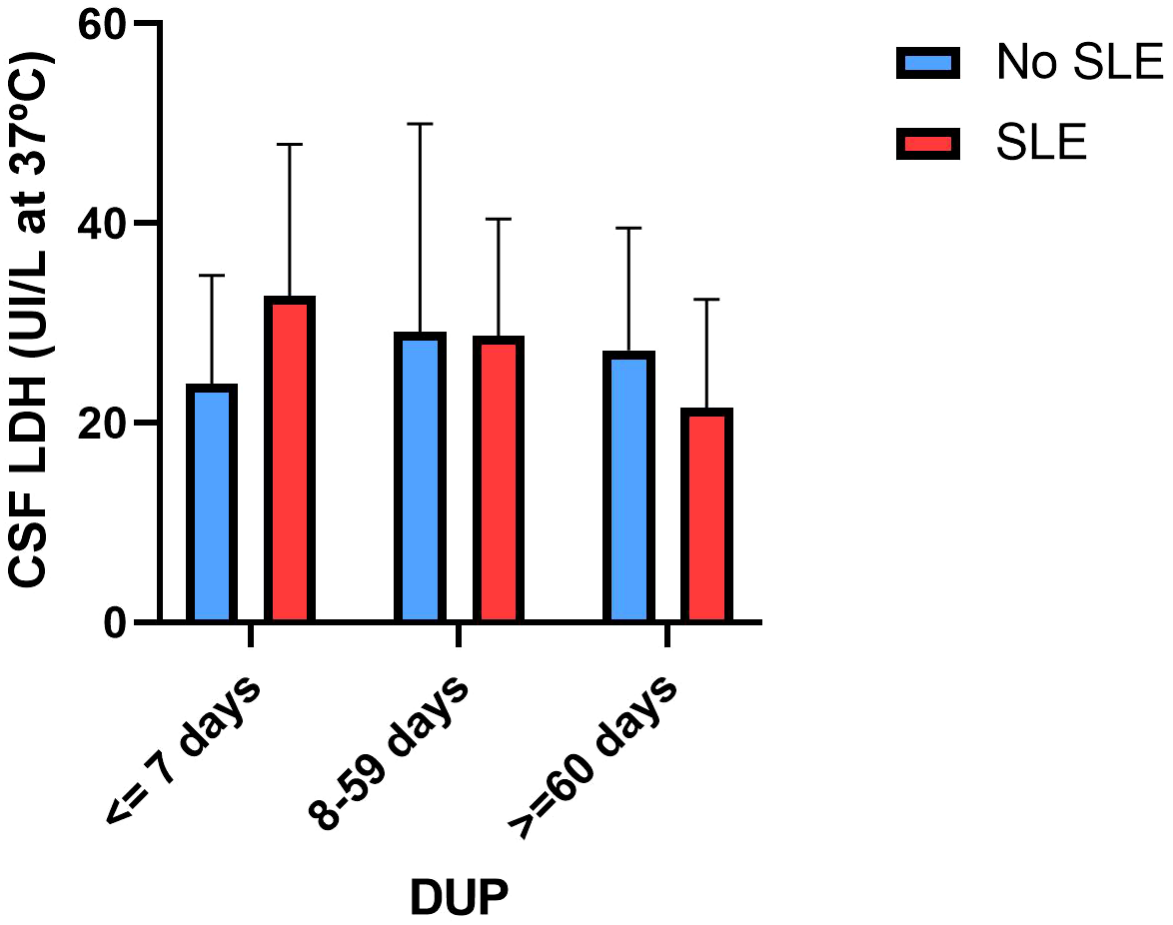
Lactate dehydrogenase concentrations in cerebrospinal fluid according to stressful life events and the duration of untreated psychosis.

In the multiple linear regression analyses, we found a significant DUP by SLE interaction effect on CSF LDH concentrations (standardized beta= -0.320, t= -2.084, p= 0.040; **Table 2**). This interaction is described in **Figure 1**: in patients with SLEs, a shorter DUP was associated with higher CSF LDH concentrations and vice versa. No significant associations were found between the DUP or SLEs and other CSF biomarkers (glucose, total proteins).

**Table 2:** Results of the multiple linear regression analysis for cerebrospinal fluid levels of lactate dehydrogenase.

Model		Unstandardized coefficients		Standardized coefficients	t	Sig.
		B	SE	Beta		
1	Constant	2.989	0.155		19.340	<0.001
	Female sex	0.004	0.117	0.004	0.034	0.973
	Age	0.005	0.004	0.143	1.332	0.186
	SLE	0.041	0.118	0.037	0.348	0.729
	DUP	-6.975E-05	0.001	-0.007	-0.061	0.951
2	(Constant)	3.040	0.154		19.777	<0.001
	Female sex	-0.041	0.117	-0.036	-0.350	0.727
	Age	0.002	0.004	0.064	0.574	0.567
	SLE	0.272	0.160	0.245	1.697	0.093
	DUP	0.001	0.001	0.131	1.061	0.292
	DUP × SLE interaction	-0.006	0.003	-0.320	-2.084	0.040

SE, standard error; SLE, stressful life events; DUP, duration of untreated psychosis.

## 4 Discussion

In our study that included 95 FEP patients, we found a significant interaction DUP by SLE interaction effect on CSF LDH concentrations. No significant associations were found between the DUP or SLEs and other CSF biomarkers (glucose, total proteins). However, it is important to note that most patients had CSF biomarkers within the normal range, and <10% of participants had abnormal CSF indices.

Our findings indicate that psychosocial stress moderates the relationship between DUP and CSF LDH concentrations. In patients with a shorter DUP, the presence of an SLE around the onset of psychosis is associated with higher CSF LDH concentrations, whereas in those patients with a longer DUP and SLEs, lower CSF LDH concentrations are found. LDH is a ubiquitous enzyme that catalyzes the interconversion of pyruvate and lactate, representing the intersection of key pathways of energy metabolism. A major concept in brain metabolism is the astrocyte-to-neuron lactate-shuttle hypothesis, which posits that astrocytes synthesize lactate through the action of LDH, which is then transported into adjacent neurons as a metabolic substrate under physiologic or pathologic conditions. Once inside the neuron, lactate is converted again by LDH to pyruvate, which then enters the mitochondrion to feed into the tricarboxylic acid (TCA) cycle. Through oxidative phosphorylation within the mitochondrial respiratory chain (MRC), adenosine triphosphate (ATP) is produced and released into the cytosolic compartment. According to a model based on refractory epilepsy, neuronal excitability can be dampened by interference with the astrocyte- to-neuron lactate shuttle: higher LDH levels are conducive to increased pyruvate levels, and higher ATP levels can lead to potassium channel inhibition, rendering the neuron more excitable (47). In a previous study from our group (35), we found that lower CSF LDH concentrations were associated with a more severe phenotype in terms of more severe prodromal symptoms and social withdrawal. Few studies have explored the role of stress in LDH activity. The SLE × DUP interaction effect on CSF LDH concentrations is intriguing. The DUP may reflect different psychotic phenotypes (FEP patients with a shorter DUP exhibiting an acute onset of psychosis and those with a longer DUP exhibiting a more insidious onset of psychosis) that involve different pathophysiological mechanisms regarding brain energy metabolism and LDH concentrations. Alternatively, the presence of psychotic symptoms and psychosocial stress might affect some CSF biomarkers (e.g., LDH) in a different way if this combination (stress and psychosis) occurs over a shorter period of time (more acute exposure) or over a longer period of time (more chronic exposure). Exposure to different stressors might activate various biological processes that have adaptive allostatic functions (48). The sustained activation of these processes, known as the “allostatic load” (AL), can potentially be harmful, potentially resulting in the onset of chronic health issues (49). Therefore, the active process of responding to a challenge to the body by triggering chemical mediators of adaptation (HPA, autonomic, metabolic, immune activity) can be adaptive in the short term (allostasis) and maladaptive in the long term (allostatic load) (48). Although speculative, we wonder whether the relationship between SLE, DUP and LDH might be explained in part by this model (adaptative responses in the short term; maladaptative responses in the long term). In those FEP patients with a shorter DUP, which would mean a more acute onset of psychosis, higher CSF LDH concentrations were found in people reporting SLEs in the previous months of the psychotic outbreak. However, in FEP patients with a longer DUP, suggesting a more insidious onset, the opposite finding was found (lower CSF LDH concentrations were found in patients with SLEs). Our speculative explanation would be that in people with psychosocial stress, higher CSF LDH concentrations represent an adaptative response but that when psychotic symptoms and SLEs last over time (equivalent to chronic stress and greater allostatic load), the reduction of CSF LDH concentrations would be a maladaptative response to stress. It is important to note that this discussion is highly speculative due to a lack of research regarding LDH and psychosocial stress based on the allostatic load model, in contrast to other HPA axis or immune biomarkers

Previous studies have established associations of serum biomarkers related to inflammatory (50–52), oxidative stress (53), immune (54), neuroendocrine (55), and metabolic systems (56) with the DUP. To our knowledge, the current study is the first to report that a CSF biomarker related to brain bioenergetics is correlated with the DUP. As the DUP is a predictor of poor clinical outcome in FEP patients (3, 4) and is associated with social withdrawal (57), future longitudinal studies might explore whether brain bioenergetics biomarkers mediate the relationship between the DUP and social functioning in the years following psychotic onset.

Our study has several limitations that need to be acknowledged. First, the project was initially designed to study autoimmunity in the CSF and serum, and our study is a secondary analysis, as we had available information on routine CSF biomarkers that we did not explore in our previous study (46). However, the original project was not designed to control for factors that could affect the bioenergetics system (such as dietary habits or fasting lumbar puncture data). Second, the FEP patients were receiving antipsychotic treatment and were not drug-naïve. However, we aimed to reduce any long-term treatment effects by excluding patients who had received antipsychotic treatment for a period longer than 6 weeks.

In summary, our study suggests that psychosocial stress moderates the relationship between the onset of psychosis and CSF biomarkers related to bioenergetic systems.

## Data availability statement

The raw data supporting the conclusions of this article will be made available by the authors, without undue reservation.

## Ethics statement

The studies involving humans were approved by Hospital of Mataró, Barcelona, Spain; Institute Ethics Committee number: 1/18. The studies were conducted in accordance with the local legislation and institutional requirements. Written informed consent for participation in this study was provided by the participants’ legal guardians/next of kin.

## Author contributions

EG-S: Conceptualization, Data curation, Formal analysis, Investigation, Methodology, Project administration, Software, Validation, Writing – original draft, Writing – review & editing. EB-Q: Investigation, Methodology, Project administration, Writing – review & editing. ED-L: Investigation, Methodology, Writing – review & editing. SC: Investigation, Methodology, Writing – review & editing. LM-S: Data curation, Investigation, Writing – review & editing. MN: Data curation, Software, Writing – review & editing. RH-A: Investigation, Methodology, Writing – review & editing. MC: Conceptualization, Formal analysis, Methodology, Supervision, Validation, Writing – review & editing. JL: Funding acquisition, Investigation, Methodology, Resources, Supervision, Validation, Writing – original draft, Writing – review & editing.
