# Corrigendum: Psychosocial stress moderates the relationship between cerebrospinal fluid lactate dehydrogenase and the duration of untreated psychosis in first-episode psychosis

**DOI:** 10.3389/fpsyt.2024.1398682

**Published:** 2024-05-23

**Authors:** Eloi Giné-Servén, Ester Boix-Quintana, Eva Daví-Loscos, Sandra Cepedello, Lara Moreno-Sancho, Marta Niubó, Rebeca Hernández-Antón, Manuel J. Cuesta, Javier Labad

**Affiliations:** ^1^ Department of Psychiatry, Hospital Universitario de Navarra, Pamplona, Spain; ^2^ Instituto de Investigación Sanitaria de Navarra (IdiSNA), Pamplona, Spain; ^3^ Department of Mental Health and Addictions, Consorci Sanitari del Maresme, Mataró, Spain; ^4^ Translational Neuroscience Research Unit I3PT-INc-UAB, Institut de Innovació i Investigació Parc Taulí (I3PT), Institut de Neurociències, Universitat Autònoma de Barcelona, Sabadell, Spain; ^5^ Centro de Investigación Biomédica en Red de Salud Mental (CIBERSAM), Madrid, Spain

**Keywords:** psychosis, duration of untreated psychosis, stress, cerebrospinal fluid, lactate dehydrogenase, glucose

In the published article there was an error in the **Introduction**. The authors sincerely apologize to Dr. Jai Shah and his group (Shah JL, Paquin V, McIlwaine SV, Malla AK, Joober R, Pruessner M. Examining the psychobiological response to acute social stress across clinical stages and symptom trajectories in the early psychosis continuum. *Dev Psychopathol* (2023) 28:1-13. doi: 10.1017/S0954579423000056) for the overlap, with missing attribution, between our article and theirs. This was an oversight which the authors deeply regret. The text previously read:

“Evidence for this diathesis has been gathered from various approaches (18), encompassing general epidemiology and prospective cohort studies (19–21), adverse childhood experiences (22, 23), clinical assessments documenting sensitivity to stress (24, 25), analyses of perceived (subjective) stress in patients (26, 27), and research on dopamine or hypothalamic–pituitary–adrenal (HPA) axis dysregulation (28–30).

In this manner, it is thought that early-life adversity and stress can interact with innate and acquired neurobiological factors to play a role in distress and, ultimately, the development of psychotic syndromes (28, 31).”

The corrected text appears below:

“Previous studies suggest that early life stress, particularly childhood trauma (18, 19), is a risk factor for psychotic disorders. Several studies have also reported increased perceived stress in individuals at clinical high risk of psychosis (CHR) or with psychotic disorders, measured using self-report questionnaires (20-22) or electronic sampling methods (23). Regarding hypothalamic-pituitary-adrenal (HPA) axis measures, previous longitudinal studies suggest that diurnal cortisol (24, 25), an increased cortisol awakening response (CAR) (26), and a higher stressor-cortisol concordance (27) are found in CHR individuals who will later develop psychotic symptoms. However, in first episode psychosis (FEP) patients, a blunted CAR has been described (28-30), suggesting that biological markers of stress response might be influenced by the stage of psychotic illness. Consistent with this, previous studies exploring stress responsiveness with the Trier Social Stress Test in CHR and FEP patients (31) found differences in autonomic measures between groups (elevated heart rate and blood pressure were observed in FEP patients), suggesting that the stage of illness contributes to variations in the psychobiological stress response.”

In the published article there was an error in the **References**. Following the correction to the **Introduction**, the below references have been added and the reference list renumbered:

The authors apologize for this error. This update does not change the scientific conclusions of the research in any way.

18. Radua J, Ramella-Cravaro V, Ioannidis JPA, Reichenberg A, Phiphopthatsanee N, Amir T et al. What causes psychosis? An umbrella review of risk and protective factors. *World Psychiatry* (2018) 17:49–66. doi: 10.1002/wps.20490

19. Arseneault L, Cannon M, Fisher HL, Polanczyk G, Moffitt TE, Caspi A. Childhood trauma and children’s emerging psychotic symptoms: A genetically sensitive longitudinal cohort study. *Am J Psychiatry* (2011) 168:65–72. doi: 10.1176/appi.ajp.2010.10040567

20. Studerus E, Ittig S, Beck K, Del Cacho N, Vila-Badia R, Butjosa A et al. Relation between self-perceived stress, psychopathological symptoms and the stress hormone prolactin in emerging psychosis. *J Psychiatr Res* (2021) 136:428–34. doi: 10.1016/j.jpsychires.2020.06.014–

21. Ortega L, Montalvo I, Monseny R, Burjales-Martí MD, Martorell L, Sanchez-Gistau V et al. Perceived stress, social functioning and quality of life in first-episode psychosis: A 1-year follow-up study. *Early Interv Psychiatry* (2021) 15:1542–50. doi: 10.1111/eip.13092

22. Ortega L, Montalvo I, Monseny R, Vilella E, Labad J. Perceived stress mediates the relationship between social adaptation and quality of life in individuals at ultra high risk of psychosis. *Early Interv Psychiatry* (2019) 13:1447–54. doi: 10.1111/eip.12791

23. Lataster T, Valmaggia L, Lardinois M, van Os J, Myin-Germeys I. Increased stress reactivity: a mechanism specifically associated with the positive symptoms of psychotic disorder. *Psychol Med* (2013) 43:1389–1400. doi: 10.1017/S0033291712002279

24. Cullen AE, Fisher HL, Gullet N, Fraser ER, Roberts RE, Zahid U, et al. Cortisol levels in childhood associated with emergence of attenuated psychotic symptoms in early adulthood. *Biol Psychiatry* (2022) 91:226–35. doi: 10.1016/j.biopsych.2021.08.009

25. Walker EF, Trotman HD, Pearce BD, Addington J, Cadenhead KS, Cornblatt BA et al. Cortisol levels and risk for psychosis: initial findings from the North American prodrome longitudinal study. *Biol Psychiatry* (2013) 74:410–7. doi: 10.1016/j.biopsych.2013.02.016

26. Labad J, Stojanovic-Pérez A, Montalvo I, Solé M, Cabezas Á, Ortega L et al. Stress biomarkers as predictors of transition to psychosis in at-risk mental states: roles for cortisol, prolactin and albumin. *J Psychiatr Res* (2015) 60:163–9. doi: 10.1016/j.jpsychires.2014.10.011

27. Cullen AE, Addington J, Bearden CE, Stone WS, Seidman LJ, Cadenhead KS, et al. Stressor-cortisol concordance among individuals at clinical high-risk for psychosis: Novel findings from the NAPLS cohort. *Psychoneuroendocrinology* (2020) 115:104649. doi: 10.1016/j.psyneuen.2020.104649

28. Cullen AE, Labad J, Oliver D, Al-Diwani A, Minichino A, Fusar-Poli P. The translational future of stress neurobiology and psychosis vulnerability: A review of the evidence. *Curr Neuropharmacol* (2024) 22:350–77. doi: 10.2174/1570159X21666230322145049

29. Labad J. The role of cortisol and prolactin in the pathogenesis and clinical expression of psychotic disorders. *Psychoneuroendocrinology* (2019) 102:24–36. doi: 10.1016/j.psyneuen.2018.11.028

30. Pruessner M, Boekestyn L, Béchard-Evans L, Abadi S, Vracotas N, Joober R, et al. Sex differences in the cortisol response to awakening in recent onset psychosis. *Psychoneuroendocrinology* 2008 33:1151–4. doi: 10.1016/j.psyneuen.2008.04.006

31. Shah JL, Paquin V, McIlwaine SV, Malla AK, Joober R, Pruessner M. Examining the psychobiological response to acute social stress across clinical stages and symptom trajectories in the early psychosis continuum. *Dev Psychopathol* (2023) 28:1–13. doi: 10.1017/S0954579423000056

